# Unraveling determinants of integrated farming systems adoption for sustainable livelihood and dietary diversity

**DOI:** 10.3389/fnut.2024.1264658

**Published:** 2024-02-09

**Authors:** K. J. Raghavendra, Jacob John, D. Jacob, T. Rajendran, A. K. Prusty, Meraj Alam Ansari, Natesan Ravisankar, Sunil Kumar, Raghuveer Singh, Mohammad Shamim, Peyush Punia, Nirmal, Amrit L. Meena, Poonam Kashyap, G. P. Shivaswamy, D. Dutta

**Affiliations:** ^1^ICAR-Indian Institute of Farming Systems Research, Meerut, India; ^2^AICRP-IFS, Kerala Agriculture University, Thiruvananthapuram, India; ^3^AICRP-IFS, Tapioca and Castor Research Station, Tamil Nadu Agricultural University, Salem, India; ^4^Southern Regional Station, ICAR-NDRI, Bengaluru, India

**Keywords:** coarsened exact matching, dietary diversity, farming systems approach, impact assessment, farm income

## Abstract

**Introduction:**

Over the years, smallholder farmers have faced more vulnerability to risk and uncertainty in India due to their dependence on cereal crops. One way to reduce this risk is through diversified agriculture, integrating different practices for efficient resource utilization, and adopting a farming systems approach. An integrated farming system (IFS) is one such technique that provides year-round income from different components of enterprises. However, the decision to adopt IFS may be determined by several characteristics of farmers, which needs to be delineated through impact analysis to harness the benefits of a systems approach.

**Methods:**

This study analyzes the economic effects of integrated farming systems and assesses their determinants, as well as the dietary diversity patterns of farmers in two states of southern India, i.e., Kerala and Tamil Nadu. A multistage sampling technique was used to obtain cross-sectional data from 367 farmers randomly chosen from one district in Kerala and two districts in Tamil Nadu. The participants have Crop + Horticulture + Animal husbandry (45.45%) as their major system, whereas non-participants have Crop + Animal husbandry (44.35%) as their predominant system. Coarsened exact matching and logit regression methods were used to evaluate the economic impacts of IFS and its influencing factors.

**Results:**

The findings of the study indicate that age, education, livestock holding, access to credit, and plantation area have a positive and significant effect on participation by farmers in the program. The matching results show that adoption of IFS resulted in a significant economic impact, generating an additional gross income of Rs. 36,165 ha^−1^ and a net income of Rs. 35,852 ha^−1^ and improving the dietary diversity of farm households by 8.6% as compared to non-adopters.

**Discussion:**

This study suggests that IFS is a promising approach for improving farmers' livelihoods, economic gains, and nutritional security. Therefore, the integrated farming systems models need to be upscaled through the convergence of government schemes in other regions of India to support smallholder farmers' farming.

## 1 Introduction

In India, nearly 60% of the population is dependent on agriculture for their livelihood. In the post-green revolution era, small and marginal farmers in India, who have predominantly focused on cereal-based production systems (1), are also contending with increased climate anomalies like floods and droughts (2). Due to these events occurring frequently, farmers are facing suboptimal agricultural production and are unable to get sufficient income to sustain their livelihood (3). The rising cost of inputs, depleting soil and water resources, land fragmentation, and imbalanced use of fertilizer and chemicals have led to many problems in agriculture production systems (4). All factors pose a severe threat to the socio-economic and environmental sustainability of agriculture. Thus, there is a need to meet the increasing demand for food from the current resources only due to the increasing population without affecting the environmental factors. Modern input-intensive, specialized agriculture affects agricultural land due to the intensification of the production system, which uses a high degree of external input as well as the incessant use of fertilizers and chemicals, causing much more damage to the flora and fauna population in the diverse agroecosystem (5, 6). With the reduction in agroecology diversity, the vulnerability due to climate change and market fluctuations increases. Even a little variation in rainfall distribution may also cause severe damage to crop establishment (7, 8). These abiotic risks in agriculture affect all categories of farmers in certain ways, but the impacts are more on marginal and small farmers (9).

In the Indian subcontinent and many other tropical countries, integrated farming systems (IFS) are followed, which are traditionally mixed animal-crop systems that have synergy between crop cultivation and livestock rearing by utilizing the by-products of each other's components for producing a greater good (10, 11). As a classical definition, IFS has been defined as a type of mixed farming system that allows crop and livestock enterprises to complement one another to maximize income and potentially minimize the risks of farmers (12, 13). The primary objective of IFS is to maintain a cyclically sustainable production system where the outputs of a specific enterprise can be used as inputs for another system within the farm (10, 11). By incorporating additional enterprise practices like crop rotation, residue management, and diversification, we can reduce the input cost and improve our income level (14). Often, crop rotation with legumes in IFS reduces the need for nitrogen fertilizer purchase, and the addition of on-farm generated farm wastes adds organic matter, which improves soil organic carbon and enhances the yield of succeeding crops (15, 16). In this way, it reduces GHG emissions and soil erosion, thereby helping to achieve greater resilience to climate change. IFS, through different combinations of crop-livestock enterprises, diversifies the systems with many crops and animal units. Diversification in agriculture is not only a measure of improved income (17) but also another dimension to combat nutritional security through diversity in the diet in rural areas (18–20). Kumar et al. (21) developed IFS models suitable for different agro-climatic regions, suggesting that all these models ensure providing food and nutritional security to farm families without fail. Diversity in consumption is important to sustain the health and nutrition of the rural population, which in turn depends on farm production diversity (22).

Recognizing the various determinants influencing the adoption of IFS technology is crucial for formulating effective policies and ensuring successful implementation. Numerous studies have pointed out that factors like education, landholding, age, and information-seeking behavior positively influence the adoption of IFS (23, 24). There are several studies linking integrated farming with dietary diversity in recent agriculture-nutrition frameworks. These pathways include factors such as farm income, market access, education of households, land holding, off-farm income, and farm diversity, as reported by Islam et al. (25) and Khandoker et al. (22). Numerous studies have been conducted in sub-Saharan Africa, particularly in the context of subsistence farming. While it's plausible that the strength of association may vary by context, further studies from Asian settings are needed to confirm this hypothesis.

For the betterment of farmers' livelihoods, Indian states like Kerala and Tamil Nadu initiated schemes to promote the IFS across the districts by providing financial and technical backstops through respective departments, especially to economically weaker sections. The integrated farming systems scheme in Tamil Nadu was started in 2018 to help farmers sustain food security (26) through enhanced productivity and income in rural areas. Similarly, in Kerala, in 2018, after severe floods in the state, it was decided to develop resilience in farming against natural calamities through the Rebuild Kerala program by implementing and popularizing IFS among the community. The government aims to achieve production holistically and sustainably because of the small size of land holdings and the homestead nature of farming in Kerala. The selected participants in the program were provided with training in integrating different enterprises and managing livestock, poultry, fisheries, and allied sectors. In light of the background, this study attempts to evaluate the determinant factors of the adoption of development schemes by state governments that promoted IFS and its impact in terms of income and dietary diversity to bridge the gap in technology adoption.

## 2 Materials and methods

### 2.1 Study area

**Figure 1:**
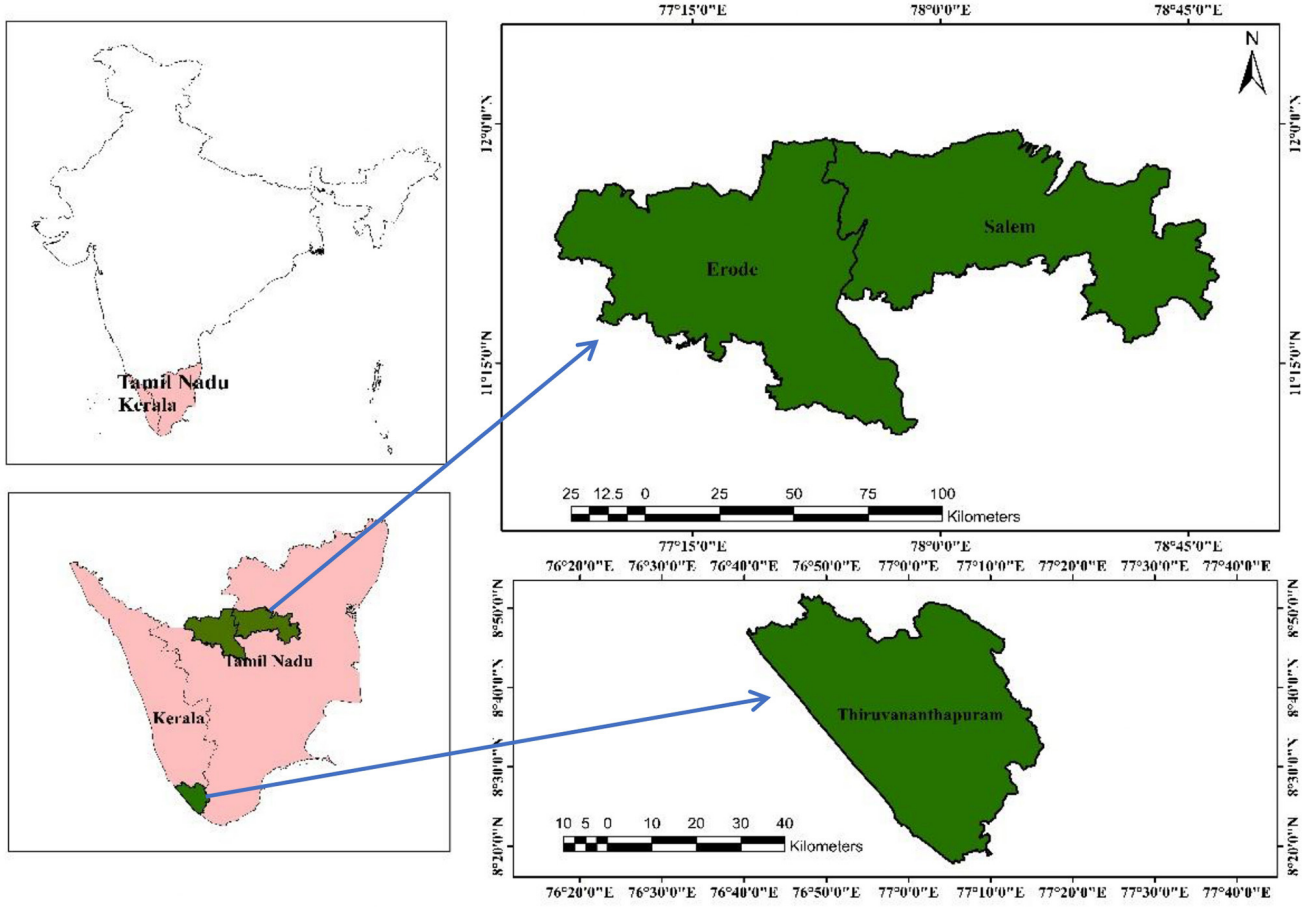
Location of the study region in India.

The South Indian states of Kerala and Tamil Nadu were selected to evaluate the impact of said program on farmers' livelihoods. The states have individually taken up schemes to promote the IFS among the farmers to elucidate its benefits and provide financial assistance to farmers. After the successful implementation of these programs in 2017 across the two states, a study was undertaken to quantify the economic benefits achieved by farmers by adopting IFS and other social impacts on their livelihoods. This study uses data from a primary survey conducted in 2021 in one district in Kerala, i.e., Thiruvananthapuram, and two districts in Tamil Nadu, i.e., Erode and Salem (Figure 1).

### 2.2 Climate of the study locale

Thiruvananthapuram is in the agro-climatic zones of the west coast plain and hills, and Salem and Erode fall in the region of the southern plateau and hills. Rainfall receipts during the monsoon in Kerala and Tamil Nadu showed remarkable variation that sets these two states apart (Table 1). The second fortnight of May marks the arrival of the southwest monsoon in Kerala, bringing torrential rain that lasts into June. However, the northeast monsoon predominates in Tamil Nadu, which receives the bulk of its monsoon rainfall between October and November. Thiruvananthapuram, Kerala, recorded a mean annual rainfall of 2,134 mm, while Salem and Erode, both in Tamil Nadu, experienced 782 mm. Rainfall receipts are subject to greater variance, with a coefficient of variation ranging from 80% in Kerala in May to 99% in Tamil Nadu in August. Farmers relying on these unpredictable monsoon rains often encounter risk and uncertainty while sowing and growing crops. Recycling organic biomass in integrated farming systems contributes to *in-situ* soil moisture conservation and nutrient recycling, lowering crop loss, which helps to offset such risks.

**Table 1:** Monsoon in sampled districts of Kerala and Tamil Nadu during 2019–2021.

	**May**	**June**	**July**	**Aug**	**Sep**	**Oct**	**Nov**	**Annual**
**Thiruvananthapuram, Kerala**								
Mean rainfall (mm)	451.23	242.77	183.17	203.05	393.40	263.79	186.70	2134.67
CV %	80.46	47.55	48.00	55.35	50.65	31.12	69.77	14.79
**Erode and Salem, Tamil Nadu**								
Mean rainfall (mm)	71.80	19.50	40.83	103.10	77.10	157.83	180.67	782.73
CV %	7.46	20.35	94.41	99.43	72.86	69.18	47.19	8.66

### 2.3 Sampling methodology

The total sample size of agricultural households is 367, out of which 193 were kept in the treatment group and 174 belonged to the control group. The samples were randomly selected in the designated district in proportion to the size of the beneficiary population. Then, the farmers were randomly selected from the sample frame available for the beneficiary category who availed of the benefit under the scheme. A sample of 174 was selected randomly with the same socio-economic condition as a control group who did not avail of any benefit under the scheme. The survey collected information on household characteristics, such as family size, age, education of the household head, social participation, resource endowment, cropping systems, livestock, and information related to economic status and household consumption. These data also provide detailed information related specifically to evaluating the impact of the farming system on returns and consumption diversity, thus being particularly suited for our analysis.

### 2.4 Analytical framework

The betterment of farmers' livelihoods needs regular income throughout the year with the ability to absorb climatic shocks like floods and droughts. IFS is one such technique that provides regular income from different components of enterprises and reduces fertilizer and chemical use, supplying healthy food for farm families (14, 27). IFS also ensures diversity in the diet through the inherent capacity of a diversified cropping system, which is a crucial element of a balanced diet.

The decision to adopt IFS may be determined by several characteristics of farmers, like land holding size, socio-economic characteristics, and their perception of the inherent features of the practices. Farmers' education, asset ownership, capacity enhancement activities, and profit-oriented behavior are the key determinants in enhancing the adoption of integrated farming systems. Also, the diversity in food consumption among farm families is analyzed. To assess the impact of technology, a researcher should be able to assess the situation in counterfactual and non-counterfactual scenarios, and inferences can be drawn and implemented as policy (28).

The quantifiable impact of IFS on income and dietary diversity of farmer households was examined using coarsened exact matching and determinants of their adoption of technology using the logit regression method.

#### 2.4.1 Household dietary diversity score

This measure is widely accepted to record the food groups consumed by households over a recall period (29). FAO (30) proposed a 12-food group, viz, cereals; white tubers and roots; legumes, nuts, and seeds; vegetables; fruits; fish and other seafood; meat; eggs; milk and milk products; oils and fats; sweets; and spices, condiments, and beverages. In this study, the first nine food groups consumed by households were considered for making HDDS healthy food groups, leaving oils and fats, sweets, and spices (22). Each food group adds one score point toward the HDDS if a food item from that group is consumed by any member of the household in the last 24 h. Thus, in this study, the HDD score ranges from 0 to 9.

#### 2.4.2 Logit regression method

The factors affecting the participation of the farmers in adoption were identified using the logit model. Logit is a technique in which the probability of a dichotomous outcome is related to a set of independent variables. It has been widely used to study adoption behavior. Suppose Xi represents the set of variables that influence the participation decisions of the i^th^ household. For a household, the indirect utility (Zi) derived from adoption is a linear function of k independent or explanatory variables (X). This can be stated as follows:


$$\begin{array}{l} Z_{i}=\beta_{0}+\overset{n}{\underset{i=1}{\sum }}\beta_{i}X_{ki} \end{array}$$

where β_0_ represents the intercept term and β_i_ is the coefficient associated with the explanatory variables X_ki. These factors explain the participation behavior and the probability that i^th^ household decides to adopt a certain practice.

The probability of participation is modeled as follows:


$$\begin{array}{l} P_{i}=\frac{e^{Z_{i}}}{1+e^{Z_{i}}} \end{array}$$

where P_i_ is the probability of i^th^ household's participation decision and (1- P_i_) denotes the probability that the household does not participate. In the analysis, independent variables like the age of the farmer, number of years of schooling, experience in the farming field, HH size, soil health card, and livestock holding were considered for the study.

#### 2.4.3 Coarsened exact matching

The most popular method of matching techniques is propensity score matching (PSM). The main drawback of this method is that it does not guarantee that the matched samples will be balanced concerning covariates X (31). As an alternative to PSM to overcome this limitation, Iacus et al. (32) have developed coarsened exact matching (CEM), belonging to the Monotonic Imbalance Bounding (MIB) group. CEM works in sample distributions and requires no assumptions about the process of data generation except for the usual ignorability assumptions. This method assures that the imbalance between the matched and unmatched groups will not be greater than the ex-ante choice stated by the user. Iacus et al. (32) have shown that CEM is better than other commonly used matching methods at reducing the imbalance, model dependence, estimation error bias, variance, and mean square error (33). With CEM, continuous variables are coarsened to discrete-interval data, and exact matching strata are constructed (34, 35).

Let *T*_i_ denote an indicator variable for unit i, which takes the value 1 if the i^th^ unit belongs to the treatment group and the value 0 if the i^th^ unit belongs to the control group. The observed outcome variable is given as follows:


$$\begin{array}{l} Y_{i}=T_{i}Y_{i}\left(1\right)+\left(1-T_{i}\right)Y_{i\;}\left(0\right) \end{array}$$

where *Y*_*i*_(0) is the outcome for the non-adopters of IFS.


$$\begin{array}{l} Y_{i}\;\text{(1) is the outcome for the adopters of IFS.} \end{array}$$

To estimate the impact of the technology intervention on a selected group of households, the standard ignorability assumption is that, conditional on X, the treatment variable is independent of the potential outcomes and that every treated unit receives the same treatment. A fixed causal effect is a function of the potential outcome, defined as *Y*_*i*_(1)−*Y*_*i*_(0).

The estimates for the causal effects on outcome variables can be defined as follows:


$$\begin{array}{l} SATT=\frac{1}{n_{t}}\underset{iet}{\sum }TE_{i} \end{array}$$

However, when all the units do not match, as is the case in the current study, SATT changes to LSATT, or local sample average treatment, for all treated plots, which is estimated by


$$\begin{array}{l} LSATT=\frac{1}{m_{t}}\underset{ieT^{m}}{\sum }TE_{i} \end{array}$$

where *m*_*T*_ is the number of matched treated units and *T*^*m*^ is the subset of matched treated units.

A few important variables, such as education level, social group of the household head, region of the respondent, membership in a social organization, and expenditure on seeds that determine adoption, are used for matching. The imbalance in the initial data was reduced from 0.66 to 0.62 after matching. The causal estimate was undertaken using the Stata software 14.1 version.

The study is intended to generate two significant conclusions; first, by mapping the livelihood of agriculture households from the integrated farming systems scheme through a detailed primary survey of respondents in these states, and second, by identifying the determinants of participation and the impact of the scheme on their income and consumption.

## 3 Results

### 3.1 Basic household characteristics of the respondents

#### 3.1.1 Socio-economic profile of respondents

The summary statistics of major socio-economic variables for sample farmers are provided in Table 2. The results indicate that non-adopters are more likely to have completed their primary education, whereas adopters are much more likely to have graduated (11.3%) compared to non-adopters. Among the socially disadvantaged classes in India, the scheduled caste and scheduled tribe (SC and ST) class had only a few adopters (12.5% compared to non-adopters). Adopters have a significantly higher population of milch animals (2.3 no.) than non-adopters (1.6 no.). The adopters had higher accessibility to credit facilities (37.0%) than the non-adopters (24.1%). Results also reveal that the adopters spent much less on fertilizer than the rest, since they recycled more farm waste than the others.

**Table 2:** Socio-economic profile of integrated farming system respondents in the sampled area.

**Variables**	**Participants (*N =* 193)**	**Non-participants (*N =* 174)**	**Difference (*t*-test)**	**Overall (*N =* 367)**
Age (years)	51.61 (11.14)	51.69 (12.13)	−0.09	51.65 (11.59)
Experience in farming (years)	23.13 (14.38)	25.16 (14.82)	−2.0	24.06 (14.59)
**Education level**				
Primary education (%)	15.38 (0.36)	27.64 (0.44)	−12.26 ^**^	20.64 (0.42)
Secondary education (%)	43.36 (0.50)	39.84 (0.49)	3.55	41.57 (0.50)
Matriculation (%)	11.89 (0.32)	15.45 (0.36)	−3.56	13.67 (0.34)
Graduation (%)	29.27 (0.46)	17.89 (0.38)	11.38 ^**^	23.97 (0.42)
Family size (no.)	4.21 (1.53)	4.06 (1.35)	0.15	4.14 (1.45)
**Social classes**				
a. Other backward castes (OBC) (%)	58.74 (0.49)	62.09 (0.49)	−3.35	60.29 (0.49)
b. Scheduled Caste and Scheduled Tribe (SC and ST) (%)	3.49 (0.18)	16.00 (0.37)	−12.51 ^***^	9.36 (0.26)
Membership in social institutions (%)	59.44 (0.49)	50.0 (0.50)	9.44	55.05 (0.50)
Milch animals (no.)	2.34 (2.461)	1.66 (1.80)	0.68 ^***^	2.03 (2.0)
Credit facility availed (%)	37.06 (0.48)	24.19 (0.43)	12.87 ^**^	31.08 (0.46)

Standard deviation is denoted in parentheses; ^**^5%, ^***^1% levels, respectively.

#### 3.1.2 Farming systems and economics of farm households

The land area and livestock units owned by farm households in the study area are presented in Table 3. The adopters had a greater number of farms (75), combining all three enterprises, followed by H+A (36) and C + A (37). The mean area under the C + A system is 1.24 ha, and overall, adopters had a 0.65 ha area under cultivation. TLU is higher for C+H+A systems (4.71), followed by C + A systems (3.41). Non-adopters had large farms under C + A (75) systems followed by C+H+A (38) components; the mean area under cultivation also follows a similar pattern. TLU is higher for adopters (3.89) compared to non-adopters (2.30).

**Table 3:** Farm income measures from different enterprise combinations.

**Farming enterprises**	**Farm households (no.)**	**Mean area (ha)**	**Total Livestock Units (no.)**
**Adopters**			
Crop+ animal husbandry (C + A)	57 (29.53)	1.24	3.41
Horticulture+ animal husbandry (H+A)	61 (31.61)	0.40	3.05
Crop+ horticulture+ animal husbandry (C+H+A)	75 (38.86)	0.56	4.71
Overall	193 (100)	0.65	3.89
**Non-adopters**			
Crop+ animal husbandry (C + A)	75 (43.10)	1.36	2.84
Horticulture+ animal husbandry (H+A)	48 (27.59)	0.25	1.58
Crop+ horticulture+ animal husbandry (C+H+A)	51 (29.31)	0.53	2.05
Total	174 (100)	0.76	2.30

Respective components are denoted in parentheses. Animal husbandry includes cow, buffalo, goat, sheep, and poultry.

**Figure 2:**
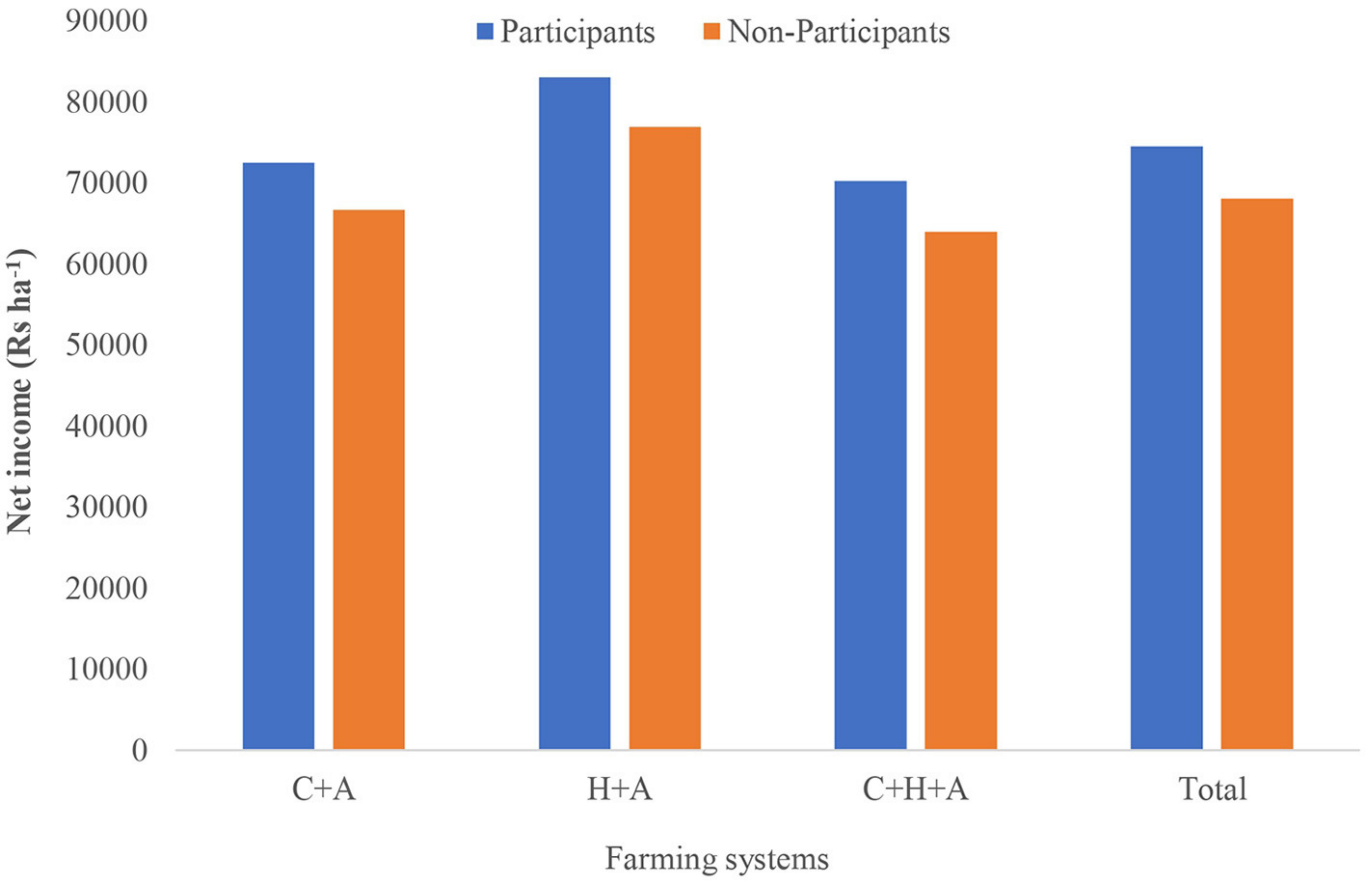
Net income from various farming systems in the study C + A: Crop + Animal husbandry; H + A: Horticulture + Animal husbandry; C + H+ A: Crop + Horticulture + Animal husbandry.

The net income of different farming systems is provided in Figure 2. In both adopters and non-adopters, H+A has a higher net return per hectare; this is due to the cultivation of high-value crops like banana, ginger, and turmeric, which yields higher returns, followed by crop + animal husbandry (C + A). The adopters have more net income compared to non-adopters in all the farming systems. In total, adopters have a net income of Rs. 74,521 ha^−1^, compared to Rs. 68,106 ha^−1^ for non-adopters of IFS.

#### 3.1.3 Consumption pattern of households

**Figure 3:**
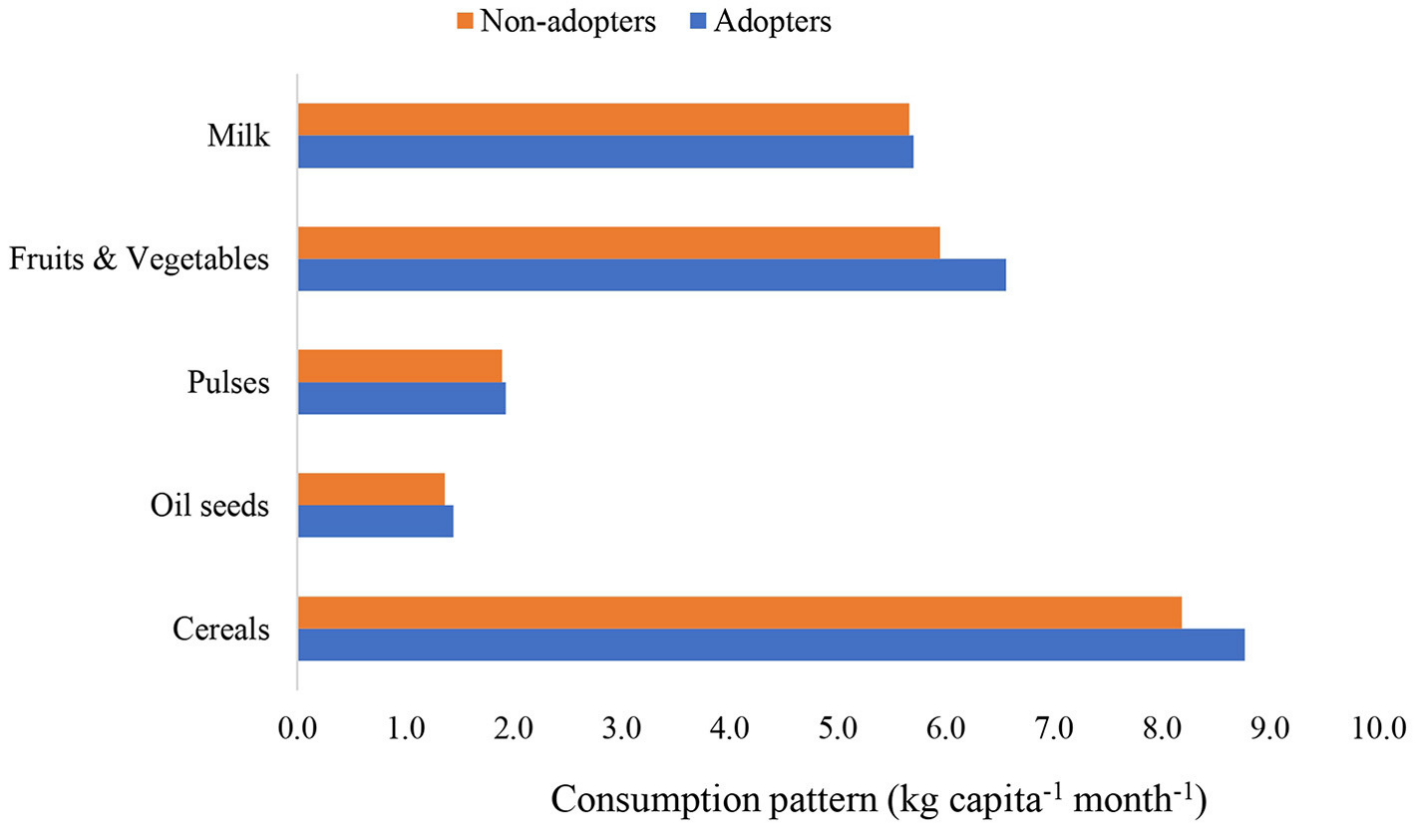
Consumption pattern of the sample households.

The patterns of consumption of major food groups are provided in Figure 3. It shows that there was a marginal increase in the consumption of cereals, fruits, and vegetables for adopters compared to non-adopters. The remaining food groups have also shown a slight improvement in consumption. Overall, in all food groups, there was a higher consumption by participants compared to others.

### 3.2 Determinants of adoption of IFS technology

The coefficient and marginal effect of logit regression estimated with observations are provided in Table 4. The results suggest that socio-demographic factors like age of respondents, education of household head, and farmers belonging to social groups SC and ST were influencing the adoption of IFS, while farming variables such as livestock holdings, the area under plantation crop, and credit facilities available were significantly and positively influencing the adoption of IFS. Agriculture households with higher education, as a proxy of knowledge, have a positive influence on the adoption of IFS compared to non-adopters. The class of farmers belonging to SC and ST was more negatively adopting integrated farming systems than others. Livestock, being an essential component aiding in the recycling of materials and utilization of fodder on the farm, positively influences the adoption of IFS. The credit facility is another important criterion that influences the adoption of IFS, as it provides financial support to invest in the adoption of different components and the utilization of available resources. Farm households with plantation crops under cultivation are more likely to adopt an integrated farming system. The increase in adoption of IFS improves by 1.8%, whereas livestock holding increases IFS adoption by 3%. Credit facilities and plantation crops improve the adoption of IFS by 12% and 11%, respectively.

**Table 4:** Determinants for the adoption of IFS among sampled households.

**Variables**	**Units**	**Marginal effect**	
		***dy/dx***	**Standard error**
Age of the respondent	Log of years	0.321 ^*^	0.160
Experience in farming	Log of years	−0.077	0.048
Education of household head	Number of years	0.018 ^**^	0.007
Household size	Numbers	0.018	0.016
Other backward caste (OBC)	Yes = 1, otherwise = 0	0.061	0.046
Scheduled Caste and Scheduled Tribe	Yes = 1, otherwise = 0	−0.335 ^***^	0.118
Landholding	Log of acres	0.028	0.004
Livestock holding	Numbers	0.033 ^**^	0.011
Plantation crops	Yes = 1, otherwise = 0	0.115 ^*^	0.061
Credit facility	Yes = 1, otherwise = 0	0.121 ^**^	0.064
Soil health card	Yes = 1, otherwise = 0	−0.155	0.126
Net income	Rs./ha	0.049	0.039
Membership in social organizations	Yes = 1, otherwise = 0	0.102	0.061
Observations		367	

^*^10%, ^**^5%, and ^***^1% levels, respectively.

### 3.3 Determinants of farm household dietary diversity

Farm household dietary diversity (HDDS) is directly connected with IFS (Table 5). The factors influencing the HDDS were assessed using the ordinary least squares (OLS) regression method. Factors like respondents' age, farmer belonging to SC and ST, land holding of the farmer, credit facility, and crops taken up in rabi had positively and significantly influenced the dietary diversity. Farmers with high farm experience have negatively influenced dietary diversity. Farm households belonging to the social class of SC and ST are more diverse in consumption compared to OBC households. Credit facilities also positively influence dietary diversity. In terms of regional effect, the households of Tamil Nadu have less diverse consumption than the households of Kerala. The crops grown in rabi positively influence the dietary diversity more than the Kharif crops. The plantation area under cultivation has a negative influence on diet diversity.

**Table 5:** Factors influencing the dietary diversity of farm households.

**Variables**	**Units**	**OLS**	
		**Coefficients**	**Standard error**
Age of the respondent	Log of years	1.084 ^***^	0.18
Experience in farming	Log of years	−0.018 ^**^	0.010
Education of household heads	Number of years	0.019	0.021
Family size	Numbers	0.058	0.051
Other backward caste	Yes = 1, otherwise = 0	−0.903 ^***^	0.195
Scheduled Caste and Scheduled Tribe	Yes = 1, otherwise = 0	0.948 ^***^	0.315
Landholding (log)	Log of Acres	0.155^***^	0.050
Livestock holding	Numbers	0.048	0.03
Crops in kharif	Yes = 1, otherwise = 0	0.059	0.050
Crops in rabi	Yes = 1, otherwise = 0	0.118 ^*^	0.062
Plantation crop	Yes = 1, otherwise = 0	−0.620 ^***^	0.215
Credit facility	Yes = 1, otherwise = 0	0.480 ^**^	0.198
Region	Yes = 1, otherwise = 0	−1.520 ^***^	0.281
Constant		2.874 ^***^	0.701
Observations		367	

^*^10%, ^**^5%, and ^***^1% levels, respectively.

### 3.4 Impact of IFS adoption on household income and dietary diversity

Results obtained from the CEM technique for four outcome variables, namely, gross income, net income, cost of cultivation, and HDDS, are provided in Table 6. The imbalance in the data has reduced from 0.71 to 0.62 after matching. ATT estimates show that the gross income of farmers who adopted integrated farming increased by Rs. 14,466 per ha in comparison to the control group. Similarly, the net income of adopters increased by Rs. 14,341 per ha in comparison to non-adopters. The cost of cultivation for adopted farmers is reduced but not statistically significant. We also measured the impact of adoptive households on HDDS. The measure has increased by 0.48 units (8.64%) in comparison to control farm households.

**Table 6:** Average treatment effect of IFS on outcome variables.

	**Control**	**Treatment**	
All	174	193	
Matched	130	150	
Unmatched	44	43	
Multivariate L1 distance: 0.62		
**Outcome variable**	**ATT**	**SE**	**Percent higher than outcome mean (%)**
Gross income (Rs/ha)	36,165^**^	17071.07	22.65
Net income (Rs/ha)	35,852 ^**^	16115.43	40.46
Cost of cultivation (Rs/ha)	−5,053 ^NS^	2151.53	-
Household Dietary Diversity Score	0.48^*^	0.25	8.64
Observations	280		

^*^10% and ^**^5% levels, respectively; ^*NS*^ non-significant; ATT, average treatment effects.

## 4 Discussion

### 4.1 Socio-economic profile of respondents

Basic household characteristics of the respondent households, including both adopters and non-adopters of IFS technology, were obtained from the survey. Significant differences between the characteristics of adopters and non-adopters were observed, implying that there is a varied level of social characteristics and structural composition in households, such as the number of milching animals, access to credit facilities, and education level, which may play a significant role in their decision for adoption or non-adoption of new technologies. For example, adopters have a large number of milching animals, better education, and access to credit compared to non-adopters.

Farm size and the progress of technology have a relationship with agricultural productivity. In the current study, the mean land-holding area of adopters was smaller as compared to non-adopters in different combinations of crop, horticulture, and animal husbandry prevalent in the study locale, which suggested that the small farm holders are more likely to adopt IFS. The findings are in agreement with the observations of Archer et al. (39), who reported that the benefits of economic prospects tend to be more pronounced for small farms as compared to large farms, and as a result, there is a higher motivation for integration in small farms. When farm sizes are small, the pressure to survive in a large population with relatively little arable land makes them very willing to adopt new technologies to increase production (40).

Furthermore, it was observed that in the overall scenario, total livestock units were higher in adopters as compared to non-adopters, which suggested the role of livestock in system integration due to the importance of manure recycling within the farm. Similar observations were reported by Akshitha and Dolli (23). Adoption of integrated farming systems fetched higher income as compared to that of non-adopters due to synergism among different components while reducing the cost of cultivation through resource recycling within the system, which is the basic principle of IFS. Adoption of IFS results in higher income in different enterprise combinations depending on the number and type of farm enterprises and their effective integration (41, 42).

In the current study among both adopters and non-adopters, H+A has a higher net return per hectare; this is due to the cultivation of high-value crops like banana, ginger, and turmeric, which yields higher returns, followed by crop + animal husbandry (C + A). Integration of horticulture into the farming system was reported to be a profitable venture and has a positive influence on the economic status of the farming community compared to its non-integration, besides improving nutritional security (43).

In the current study, increased consumption of food groups among IFS adopters was noticed as compared to non-adopters. According to Innazent et al. (44), the adoption of an integrated farming system (IFS) helps farm families achieve food and nutritional security, particularly for small and marginal holdings. The adoption of agricultural technologies can increase household food consumption (45). The importance of IFS technology in achieving food and nutritional security for households has been reported by several authors (5, 46, 47).

### 4.2 Factors influencing IFS adoption

The results showed that household-specific, socioeconomic, and institutional factors influence the adoption of integrated farming systems. The study finds that age and education influence the adoption of IFS positively. The age of respondents as a proxy for experience had a significant positive influence on IFS adoption (23, 24, 35). It is believed that education influences adoption by boosting a farmer's ability to “perceive, interpret, and respond to new events in the context of risk” (48). The results indicated that non-adopters are more likely to have completed their primary education, whereas adopters are much more likely to have graduated. This suggested that farmers with better education are earlier adopters of modern technology (49, 50). As IFS technologies require efficient integration of components, education is expected to strongly encourage the adoption of integrated farming systems. Many studies have also postulated that a higher educational level should enhance the probability of higher adoption of modern farming technology (51, 52). Education is also typically related to awareness of government programs in general, and understanding of technology for adoption is also better with education. Households who accessed the agricultural credit facility will diversify their farm enterprises more than those who did not (53, 54). Asante et al. (55) found that farmers require financial liquidity to incorporate both crops and animals, as well as other inputs (e.g., labor) and equipment for crop cultivation. Thus, credit availability improves the integration of other components in the existing system. This suggests that when credit facilities are available to households, there is a higher chance of technology being adopted. It is believed that access to credit encourages the adoption of technologies by boosting households' risk-bearing capacity (38). Livestock provides huge scope for on-farm recycling of manure and reduces fertilizer purchases, while also providing high profits per unit area of land used (56). Livestock is also a form of savings in rural areas that can easily be liquidated to bridge income gaps that may arise within a household (57). The integration of plantation components influences the adoption of IFS positively, as it reduces the cost of maintenance and also increases the market price for plantation commodities. From the plantation, a lot of raw materials in the form of fodder and trash were available, which could be recycled through IFS adoption. Similar results have been observed in some earlier studies (13, 58).

### 4.3 Factors influencing the dietary diversity of farm households

Results revealed that the age of the household head positively influenced dietary diversity. As age increases, understanding of the importance of dietary diversity improves in rural areas (59). The households belonging to SC and ST have higher dietary diversity compared to OBC households. These results are in line with Sarkar, (60), but in contrast with Bansal et al. (61), as the dietary diversity of different social castes is influenced depending on geography, social conditions, and culture. The relationship is positive for livestock integrated into IFS models, with the availability of milk and milk products influencing diverse consumption positively (37). The results showed that the farm size of a household has a positive relationship with HDDS. Aidoo et al. (62) found that this was expected because households with large farm sizes can cultivate different varieties of crops and rear livestock. The negative relationship of plantation crops with dietary diversity is due to the fact that these commercial crops need primary processing to some extent before consumption, resulting in a negative influence on dietary diversity. Credit facilities improve the financial conditions of farm households, leading to diverse crop cultivation so that they will have more diverse food consumption (63). Here, the negative relationship of the region dummy shows that Tamil Nadu farmers are less diverse in the diet in comparison to Kerala, as regional characteristics play a crucial role in the diet of households (59). The farmers in Kerala grow more high-value crops and have better economic status, leading to higher dietary diversity. Impact of IFS on household income and dietary diversity

The study was found to be positive and significant for adopters and non-adopters, indicating that IFS adoption has had a significantly positive impact on the welfare of the farmers (64). Specifically, sustainable agricultural practices through integrated farming systems adoption increase farm gross and net income by 23% and 40%, respectively. Our results are largely supported by previous studies (36, 65–68). The study also finds that the HDD score has improved by 8% for adopters compared to others. Previous studies also attributed that the integration of additional farm enterprises into the farming systems will potentially improve the diversity of household diets and nutritional outcomes (69–71).

## 5 Conclusion

The innovativeness of the study is that it addresses the important issue of enhancing farm income and dietary diversity, which are in line with the Sustainable Development Goals (SDGs) by analyzing the impact farming systems approach from a larger perspective. The primary data was collected from Kerala and Tamil Nadu, with 367 observations. The robustness of the methodology using logit and OLS regression methods to identify the influencing factors of adoption and consumption and a matching technique to study the impact on farmers' wellbeing paves the way for the usefulness of the current study in impact analysis and extending the benefits of IFS. The study found that integrated farming systems have a positive and significant impact on farmers' economic wellbeing and dietary diversity; thus, they improve the livelihoods of rural communities. The variables like age, education, social class, credit facilities, and plantation crop of the farmers influenced their adoption behavior concerning IFS. The relationship between farming system technology adoption and improved livelihood is assumed to be simple. However, quantifying the effect of technology adoption can be quite complex. The study demonstrated that IFS has a huge scope for income enhancement and the wellbeing of farmers, particularly smallholders. This article attempts to fill the gap by evaluating the outcomes of developmental schemes that adopted a farming system approach for large-scale dissemination of IFS technologies.

### 5.1 Policy implications

This study recommends that concerted efforts need to be taken to develop and promote IFS across other state governments as a viable option for sustainable food production and household nutrition security. The benefits of IFS adoption could be extended to a larger population through policies that address the constraints in the adoption of technology, like extending credit facilities, education, and awareness. Improved access to technology will increase both the spread and intensity of IFS adoption. This also needs to be accompanied by sufficient infrastructure facilities at backward and forward production linkages.

## Data availability statement

The original contributions presented in the study are included in the article/supplementary material, further inquiries can be directed to the first/corresponding authors.

## Ethics statement

The studies involving humans were approved by the Institute Research Council of ICAR-IIFSR (Project code IXX18170) and results were also presented and approved by AICRP on IFS Biennial workshop held during 18–21 January 2023 and approved by ICAR Vide F No NRM/7–10/2020-AFC dated 27 April 2023. The study involved survey of participants for data collection upon informed written consent to be part of survey. The studies were conducted in accordance with the local legislation and institutional requirements. The participants provided their written informed consent to participate in this study.

## Author contributions

KR: Conceptualization, Data curation, Formal analysis, Writing–original draft. JJ: Data curation, Writing–review and editing. DJ: Data curation, Writing–review and editing. TR: Data curation, Writing–review and editing. AP: Investigation, Methodology, Formal analysis, Writing–review and editing. MA: Investigation, Methodology, Formal analysis, Writing–review and editing. NR: Project administration, Supervision, Writing–review and editing. SK: Project administration, Supervision, Writing–review and editing. RS: Writing–review and editing, Conceptualization. MS: Writing–review and editing, Conceptualization. PP: Conceptualization, Writing–review and editing. N: Conceptualization, Writing–review and editing. AM: Writing–review and editing, Conceptualization. PK: Writing–review and editing, Data curation. GS: Formal analysis, Writing–review and editing. DD: Writing–review and editing, Formal analysis.
